# High fidelity visualization of cell-to-cell variation and temporal dynamics in nascent extracellular matrix formation

**DOI:** 10.1038/srep38852

**Published:** 2016-12-12

**Authors:** Claire M. McLeod, Robert L. Mauck

**Affiliations:** 1 Department of Bioengineering, University of Pennsylvania, Philadelphia, PA 19104, USA; 2 McKay Orthopaedic Research Laboratory, Department of Orthopaedic Surgery, Perelman School of Medicine, University of Pennsylvania, Philadelphia, PA 19104, USA; 3 Translational Musculoskeletal Research Center, Philadelphia VA Medical Center, Philadelphia, PA 19104, USA

## Abstract

Extracellular matrix dynamics are key to tissue morphogenesis, homeostasis, injury, and repair. The spatiotemporal organization of this matrix has profound biological implications, but is challenging to monitor using standard techniques. Here, we address these challenges by using noncanonical amino acid tagging to fluorescently label extracellular matrix synthesized in the presence of bio-orthogonal methionine analogs. This strategy labels matrix proteins with high resolution, without compromising their distribution or mechanical function. We demonstrate that the organization and temporal dynamics of the proteinaceous matrix depend on the biophysical features of the microenvironment, including the biomaterial scaffold and the niche constructed by cells themselves. Pulse labeling experiments reveal that, in immature constructs, nascent matrix is highly fibrous and interdigitates with pre-existing matrix, while in more developed constructs, nascent matrix lacks fibrous organization and is retained in the immediate pericellular space. Inhibition of collagen crosslinking increases matrix synthesis, but compromises matrix organization. Finally, these data demonstrate marked cell-to-cell heterogeneity amongst both chondrocytes and mesenchymal stem cells undergoing chondrogenesis. Collectively, these results introduce fluorescent noncanonical amino acid tagging as a strategy to investigate spatiotemporal matrix organization, and demonstrate its ability to identify differences in phenotype, microenvironment, and matrix assembly at the single cell level.

In tissues throughout the body, the extracellular matrix (ECM) guides cell phenotype and imparts mechanical resilience over a lifetime of load-bearing use. These extracellular matrices are highly dynamic, and change in both structure and molecular composition as development progresses, and with aging and disease processes. In articular cartilage, the ECM transitions from a fibronectin-rich environment in early development, to one dominated by aggrecan and collagen II at tissue maturity^1^. Notably, in both developing and mature cartilage, matrix synthesis and turnover occur continuously, and are requisite for tissue homeostasis^2^. Unfortunately, this homeostasis is often disturbed by injury- and degeneration-induced damage to the cartilage matrix and its resident cells. Such damage fails to intrinsically heal, and has prompted the development of engineered cartilage replacements.

In the context of cartilage tissue engineering, chondrocytes and progenitor cells must not only create matrix, but also retain and assemble it in the pericellular space. The rates of ECM production, retention, and degradation define how rapidly an engineered construct can mature. Thus, the manner in which the matrices produced by individual cells interact and integrate with one another ultimately defines the functional properties of the tissue that forms^3^,^4^. Moreover, just as the *in vivo* ECM influences cell phenotype in native tissue, the structure and composition of the matrix in these *in vitro* constructs regulates the extent and progression of chondrogenesis^5^. Thus, heightened understanding of matrix protein synthesis and remodeling is relevant to contexts spanning development, disease, and tissue engineering.

Towards the quantification of matrix dynamics, ECM formation can be monitored via bulk biochemical measures across time and disease state. However, such ensemble approaches mask cell-to-cell variation and do not provide information regarding the spatial organization of the matrix. Alternatively, autoradiography with radiolabeled sulfate and proline can provide insight into the localization of proteoglycans and collagens around individual cells, and has demonstrated temporal changes in the rate and spatial distribution of secreted matrix^6^,^7^,^8^. However, this approach is inherently complicated by its use of radioisotopes. Moreover, the punctate pattern of autoradiographic grains offers limited information regarding the structure and organization of this nascent extracellular matrix. To overcome these limitations, we introduce the use of a metabolic labeling approach, functional noncanonical amino acid tagging (FUNCAT), to enable high fidelity fluorescent observation of nascent extracellular matrix protein accumulation and assembly. Previously, FUNCAT has been used to visualize protein synthesis and intracellular trafficking in cell monolayers^9^,^10^,^11^,^12^, bacteria^13^, larval zebrafish^14^, and drosophila^15^. FUNCAT relies on “residue-specific” incorporation of non-canonical amino acids (ncAA) into proteins as they are synthesized^16^. While many ncAAs exist and collectively offer a diverse suite of functions, the ncAAs utilized in FUNCAT are restricted to those that contain bio-orthogonal functional groups that can be detected by highly selective fluorescent tags following ncAA incorporation. Operationally, FUNCAT ncAA incorporation resembles pulse labeling: a canonical amino acid (cAA) is removed from the environment, and is replaced with a corresponding ncAA^9^,^16^. In the absence of the cAA, the endogenous translation machinery of the cell incorporates the ncAA into proteins during synthesis, yielding global incorporation of the ncAA across the nascent proteome^16^. This strategy contrasts with “site-specific” ncAA incorporation, which utilizes genetic manipulation to substitute ncAAs in targeted locations, and more advanced residue-specific strategies that rely on engineered biosynthetic machinery to incorporate ncAA^16^.

In this study, we adapt the FUNCAT technique to enable the fluorescent visualization of extracellular matrix proteins in both native cartilage and in 3D engineered constructs. Our results demonstrate that the FUNCAT method enables high fidelity labeling of extracellular matrix proteins throughout the time course of matrix formation and homeostasis. We use this labeling approach to query cell-to-cell heterogeneity in matrix formation and to determine how the density of the microenvironment, crosslinking of nascent ECM proteins, and the pre-established ECM influence matrix protein distribution and assembly on a single cell basis using both primary chondrocytes and mesenchymal stem cells undergoing chondrogenic differentiation.

## Results

### Methionine analogs enable the fluorescent labeling of extracellular matrix proteins

**Figure 1:**
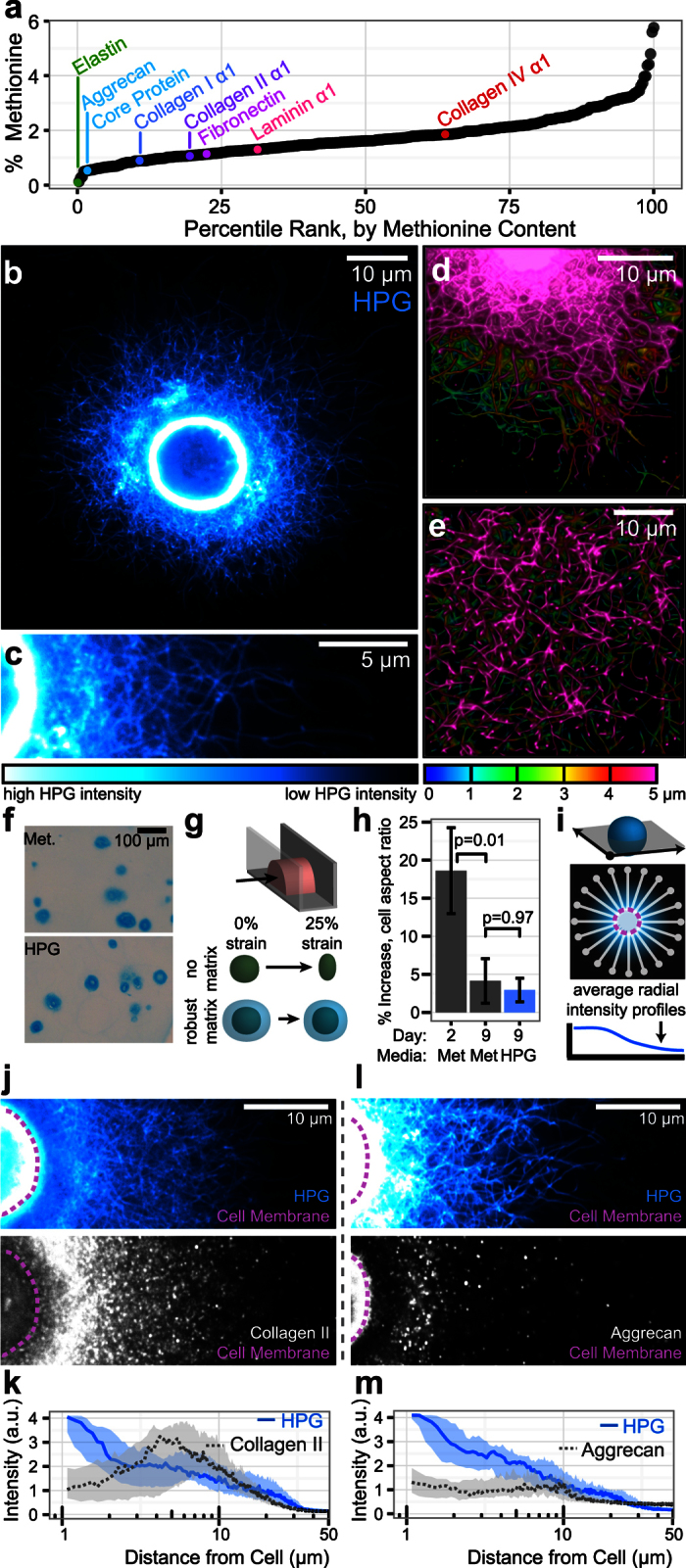
HPG labeling enables high fidelity visualization of the extracellular matrix. **(a)** Percentage methionine content of 361 extracellular matrix proteins, ordered by relative methionine abundance. **(b,c**) Confocal cross section of a chondrocyte cultured in agarose and continuously labeled with HPG for 9 days. **(d,e**) 3D reconstruction of 5 μm confocal stacks taken near the cell midplane (**d**) and below the cell (**e**) of day 9 chondrocytes. Color indicates vertical position in the stack. **(f)** Alcian blue staining of constructs cultured in control media with native methionine and labeling media containing HPG. **(g)** Schematic illustrating microcompression to assess extracellular matrix mechanics. **(h)** Percent increase in cellular aspect ratio following compression. Bars represent mean ± SEM (n = 40 cells/group, compared via ANOVA with Tukey’s post hoc test). **(i)** Schematic illustrating radial profile quantification of extracellular HPG labeling. **(j,k)** Images and radial profile quantification of simultaneous collagen II immunostaining and HPG labeling. **(l,m)** Images and radial profile quantification of simultaneous aggrecan immunostaining and HPG labeling. For (**k**) and (**m**), lines represent median intensity profile, shaded areas represent 25^th^ to 75^th^ percentiles (n = 20 cells/group).

Current implementations of FUNCAT rely on the substitution of the cAA methionine, an amino acid that comprises between 0–6% of the residues in ECM proteins (Fig. 1a, Supplementary Table S1). For example, methionine constitutes 1.1% of fibronectin and 1.8% of collagen IV, an ECM protein found primarily in basal lamina. For articular cartilage, our tissue of interest, methionine represents ~1% of the amino acid content^17^,^18^, with similar relative abundance amongst the major cartilage ECM constituents collagen II (1.08%) and aggrecan core protein (0.54%). In the present work, we separately considered labeling with two bio-orthogonal methionine analogs: homopropargylglycine (HPG) and azidohomoalanine (AHA)^10^. These analogs are structurally and functionally similar to native methionine, but include an alkyne and azide side chain respectively.

To assess the ability of HPG and AHA to identify nascent ECM proteins, we first cultured chondrocytes in thin (~400 μm thickness) 2% agarose gels for 9 days in the absence of native methionine and the continuous presence of either HPG or AHA. Following HPG or AHA culture and sample fixation, we identified incorporated HPG and AHA by labelling alkyne or azide residues with fluorescent azide or alkyne tags, respectively, via a copper-catalyzed azide-alkyne cycloaddition (“click”) reaction. This staining procedure fluorescently and covalently labeled proteins synthesized in the presence of each ncAA, and was combined with additional fluorescent staining to identify the cell nuclei as well as the cell membrane (to distinguish intracellular proteins from extracellular matrix components). Stained gels were whole-mounted and imaged via confocal microscopy at high magnification (40–100X). Scans across the surface and through the depth suggested that labeling intensity was uniform throughout the gel (Fig. S1). Individual cells exhibited extensive extracellular staining that had a clear fibrous structure (Fig. 1b–e). Near the cell midplane (Fig. 1b–d, Movie 1), densely packed HPG-tagged proteins extended outward from the cell body, forming a mesh-like structure. Labeled proteins often undulated through multiple z-planes, emphasizing the 3D nature of forming ECM. Below the cell (Fig. 1e, Movie 2), protein was more loosely organized and fibrous structures were vertically oriented towards the cell body. The labeling patterns of HPG and AHA were similar (Fig. S2), and so HPG was utilized for the remainder of the studies. Control samples cultured with native methionine instead of HPG showed minimal click reaction staining, confirming that the labeling is highly specific, with minimal off-target binding or labeling (Fig. S3).

To confirm that the substitution of methionine with HPG did not impact matrix accumulation, we compared gels cultured for 9 days in either labeling media (with HPG) or control media (with native methionine). Matrix accumulation, marked by Alcian blue staining of histological sections, was similar between the labeling and control groups (Fig. 1f). Furthermore, HPG incorporation did not alter the mechanical function of the formed matrix. We performed microcompression testing to monitor matrix stress shielding and infer the mechanical properties of the nascent ECM^19^. In this assay, cells with little or weak ECM deform readily with bulk compression of the gel, while cells with robust pericellular matrix accumulation deform much less (Fig. 1g). At day 2, cells deformed readily in response to applied strain (18.6% ± 5.6%). By day 9, cell deformation was attenuated (4.1% ± 2.9%, p = 0.01 vs Day 2), and the extent of this attenuation was nearly identical between control and labeled groups (labeled: 2.9% ± 1.5%, p = 0.97 vs Day 9 Methionine, Fig. 1h). Thus, the matrix produced in the presence of HPG was similar in both its distribution and mechanical function compared to matrix formed in standard culture conditions.

Because HPG should label all methionine-containing proteins, we next asked how the spatial pattern of HPG labeling compared with that of specific extracellular matrix proteins. For this and all subsequent studies, individual cells were identified via nuclear staining and imaged through the midplane of the cell body (Fig. 1i). Simultaneous staining for HPG and either aggrecan core protein (Fig. 1j,k) or collagen II (Fig. 1l,m) emphasized the high degree of structural detail revealed by FUNCAT in comparison with traditional staining methods. Aggrecan core protein was restricted to the pericellular space, an area where HPG labeling was often most intense (Fig. 1j,k). In contrast, collagen II co-localized with the outer reaches of HPG labeling, both qualitatively (Fig. 1l,m) and as quantified by average intensity profiles directed radially from the cell surface and extending into the gel (Fig. 1k,m). Pre-treatment of fixed samples with hyaluronidase revealed additional collagen II staining closer to the cell, but did not influence the outer radius of staining, nor did it alter the co-localization between collagen II and HPG at the matrix boundary (Fig. S4). Collectively, the tight co-localization of HPG signal with prevalent matrix proteins was consistent with the expectation that HPG would incorporate into, and label, the proteinaceous components of the extracellular matrix.

### Metabolic labeling tags pericellular collagenous network

**Figure 2:**
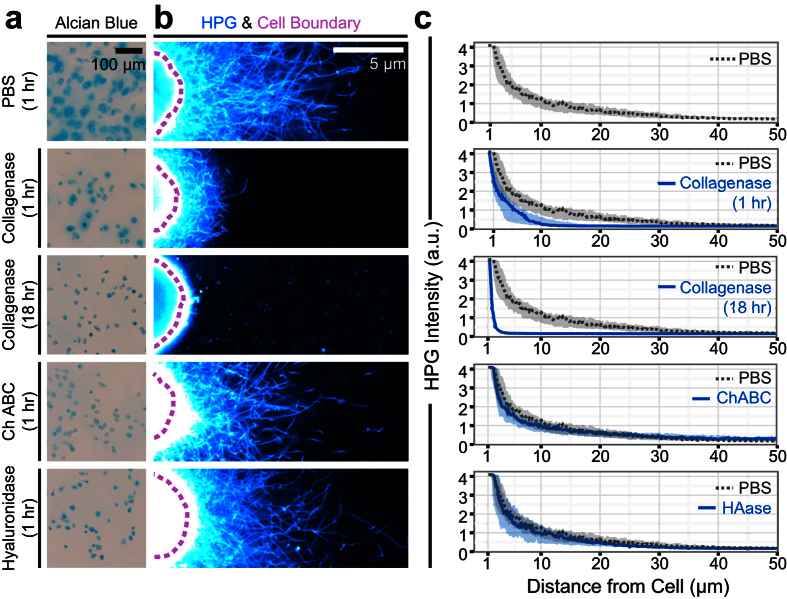
Enzymatic digestion differentially effects HPG-labeled matrix. **(a)** Alcian blue staining of chondrocyte/agarose constructs cultured and labeled with HPG for 9 days followed by enzymatic digestion prior to fixation. **(b)** Corresponding visualization of HPG-labeled matrix in digested samples. **(c)** Radial profile quantification of HPG intensity following digestion, compared to constructs incubated in PBS. Lines represent median intensity profile, shaded areas represent 25^th^ to 75^th^ percentiles (n = 20 cells/group). To highlight behavior closest to the cell, data are visualized over log (distance) in Fig. S5.

To better understand the identity and relative organization of the extracellular proteins labeled with HPG, we enzymatically digested chondrocyte-generated ECM prior to sample fixation on day 9. Alcian blue staining confirmed the efficacy of digestion with collagenase, hyaluronidase, and chondroitinase ABC (Fig. 2a). Collagenase digestion of the fibrous collagen network completely removed HPG-labeled proteins accumulated near the cell, and yielded short fibrous protein fragments that were distributed throughout the gel (Fig. 2b,c). In contrast, digestion with either hyaluronidase or chondroitinase ABC had only subtle effects on the pattern of HPG labeling (Fig. 2b,c, Fig. S5). Because hyaluronan and chondroitin sulfate are not proteins, they are not tagged directly by HPG labeling. Instead, hyaluronan digestion would be expected to disrupt pericellular proteoglycan aggregates, while chondroitinase ABC would be expected to remove the fixed negative charge density and cause a loss of charge-bound proteins in the pericellular space^20^. Thus, these results suggest that, at this time point, the majority of extracellular proteins labeled by HPG are collagens, or proteins that rely on collagens to be retained in the pericellular space.

### Biophysical features of the cellular microenvironment influence matrix organization

**Figure 3:**
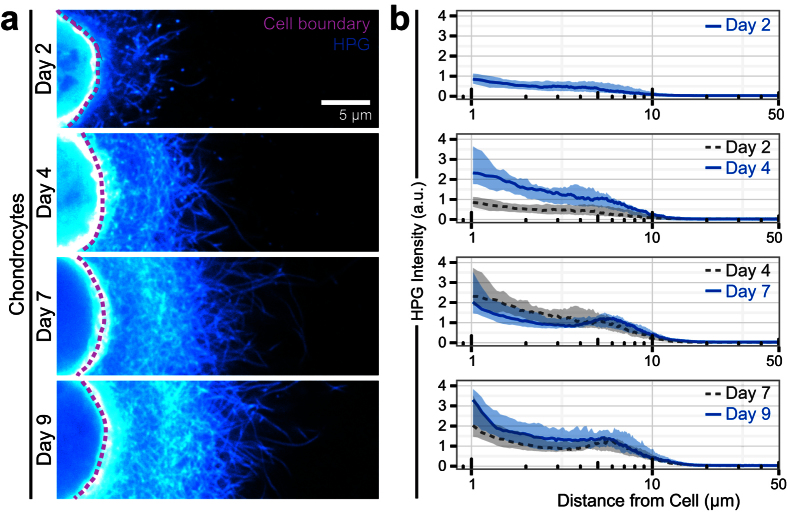
HPG labeling captures nascent matrix deposition. Images (**a**) and radial profile quantification (**b**) of chondrocytes cultured in 2% agarose and continuously labeled with HPG for up to 9 days. Colormap and scale bar are consistent across all images. Lines represent median intensity profile, shaded areas represent 25^th^ to 75^th^ percentiles (n = 20 cells/group).

To assess the ability of HPG labeling to identify differences in matrix catabolism and organization, we next used continuous HPG exposure to monitor the time course of matrix accumulation by chondrocytes. With time, extracellular proteins progressively extended from the cell (Fig. 3a,b). Matrix distributed symmetrically around individual chondrocytes (Fig. S6a), and overall labeling intensity increased with time in culture (Fig. 3a,b). By day 7, HPG labeling in chondrocytes often exhibited a low-intensity band adjacent to the cell surface, potentially reflecting the formation of a pericellular matrix rich in proteoglycans.

**Figure 4:**
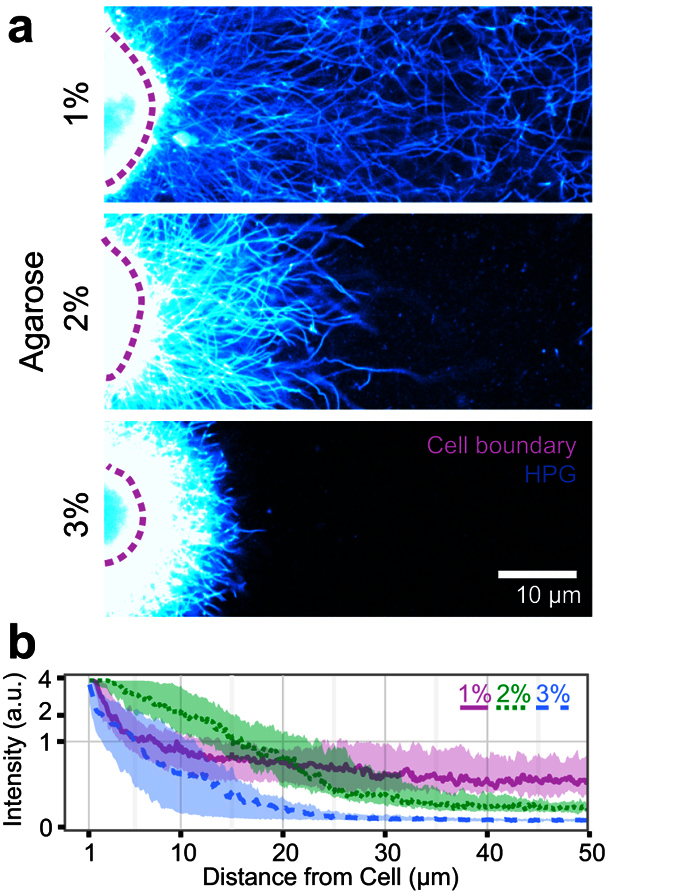
Density of the cellular microenvironment impacts organization and distribution of nascent matrix. **(a)** Images of HPG-labeled chondrocytes cultured in agarose of varying densities. **(b)** Radial profile quantification of HPG intensity in 1%, 2%, and 3% agarose hydrogels.

The structure of this accumulating matrix was modulated by the cellular microenvironment, including the choice of the biomaterial scaffold and its physical properties. We have previously demonstrated that increasing hydrogel density promotes matrix formation, but prevents matrix distribution throughout the material^3^. To examine this behavior at the single cell level, we cultured chondrocytes in 2% agarose gels (the standard condition), as well as 1% and 3% agarose gels, for 9 days. Extracellular proteins distributed readily throughout the 1% gel, resulting in a disperse protein network that extended between neighboring cells (Fig. 4a,b). Conversely, in denser 2% and 3% gels, individual cells retained discrete extracellular protein; accumulated protein was most compact in 3% agarose (Fig. 4a,b). Thus, as the microenvironment became increasingly dense and less permissive, matrix proteins were spatially restricted to their point of origin.

### Nascent matrix intersperses with pre-existing matrix in developing (but not developed) microenvironments

**Figure 5:**
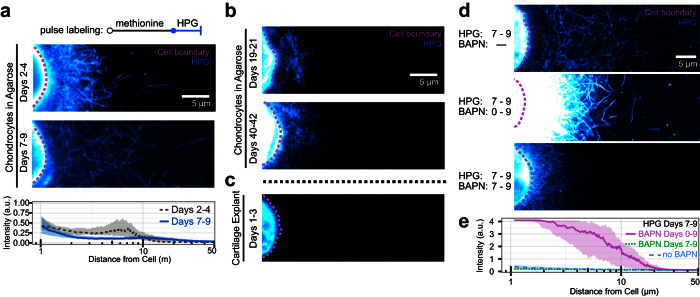
Extent and organization of nascent matrix assembly depend on construct maturity and collagen cross-linking. **(a)** Images and radial profile quantification of chondrocytes cultured in agarose for 4 and 9 days that were pulse-labeled with HPG for the final 2 days of culture. **(b)** Chondrocytes cultured in agarose for 21 and 42 days, and pulse labeled for the final 2 days of culture. **(c)** Pulse-labeled chondrocyte in a cartilage explant, cultured for 3 days. **(d)** Pulse-labeled (final 2 days of culture) chondrocytes cultured in the presence or absence of BAPN, a collagen cross-linking enzyme inhibitor. BAPN was administered either continuously or over the final 2 days of culture. **(e)** Radial intensity profiles of day 7–9 pulse labeled chondrocytes in the presence and absence of BAPN. Colormaps are consistent within subfigures. Lines represent median intensity profile, shaded areas represent 25^th^ to 75^th^ percentiles (n = 20 cells/group).

Next, we asked how proteinaceous matrix forms and is organized within the context of an existing extracellular matrix. That is, do matrix proteins accumulate in concentric rings (like growing trees), or does new matrix interdigitate with the pre-existing structure? To answer this question, gels were cultured in control media for 2, 7, 19, and 40 days and switched to HPG labeling media for the two final days of culture. Only proteins synthesized during the final two days would be labeled with HPG, while pre-existing protein would be unlabeled. Results from this study showed that, between 2 and 4 days, nascently produced fibrous proteins accumulated in the pericellular space (Fig. 5a). Fibrous structures also formed between days 7 and 9, and were distributed within the pre-existing matrix (Fig. 5a). Between 19–21 days and 40–42 days, however, nascent proteins were primarily restricted to the immediate pericellular space and lacked a fibrous structure (Fig. 5b). This likely reflects an increasingly dense microenvironment and a potential shift in the molecular composition of the nascent matrix. Cartilage explants cultured for 3 days in labeling media showed little evidence of nascent proteins in the extracellular space (Fig. 5c), suggesting that matrix turnover in mature tissue occurs on a timescale longer than the labeling period. Collectively, these results are consistent with the notion that the permissivity of the existing matrix dictates nascent matrix elaboration and organization. At early time points in culture, cells form matrix rapidly and incorporate this newly formed material within the existing matrix. As the existing matrix matures, increased matrix density restricts nascent proteins to the more immediate pericellular space.

Because matrix formation and accumulation are highly regulated processes, we also asked how perturbation of normal matrix assembly might alter nascent matrix protein synthesis and organization. To do so, we used β-aminopropionitrile (BAPN) to inhibit the collagen crosslinking enzyme, lysyl oxidase. Here, continuous exposure to BAPN for 9 days dramatically altered matrix organization, but did so in a non-intuitive manner. In the context of BAPN, continuous labeling revealed dense and intensely labeled proteins adjacent to the cell (Fig. S7). The extent of extracellular protein deposition was sharply truncated at 7.9 ± 0.5 μm from the cell, suggesting that matrix proteins were unable to extend into the hydrogel as they had in control samples. Pulse labeling with HPG from 7–9 days showed that continuous BAPN treatment (from day 0) influenced not only the spatial organization of matrix proteins, but also their synthesis dynamics. In pulse-labeled control groups, matrix protein labeling was of low intensity and interspersed within the pre-existing matrix (Fig. 5d,e; Fig. S7). Strikingly, BAPN treatment increased the amount of nascent extracellular proteins produced, and restricted these proteins to the immediate pericellular space. Intriguingly, these results suggest that collagen crosslinking is necessary for the cell to project newly formed collagen outward into the microenvironment, and for cells to achieve matrix homeostasis.

### Patterns of matrix deposition identify phenotypic differences between cell types

**Figure 6:**
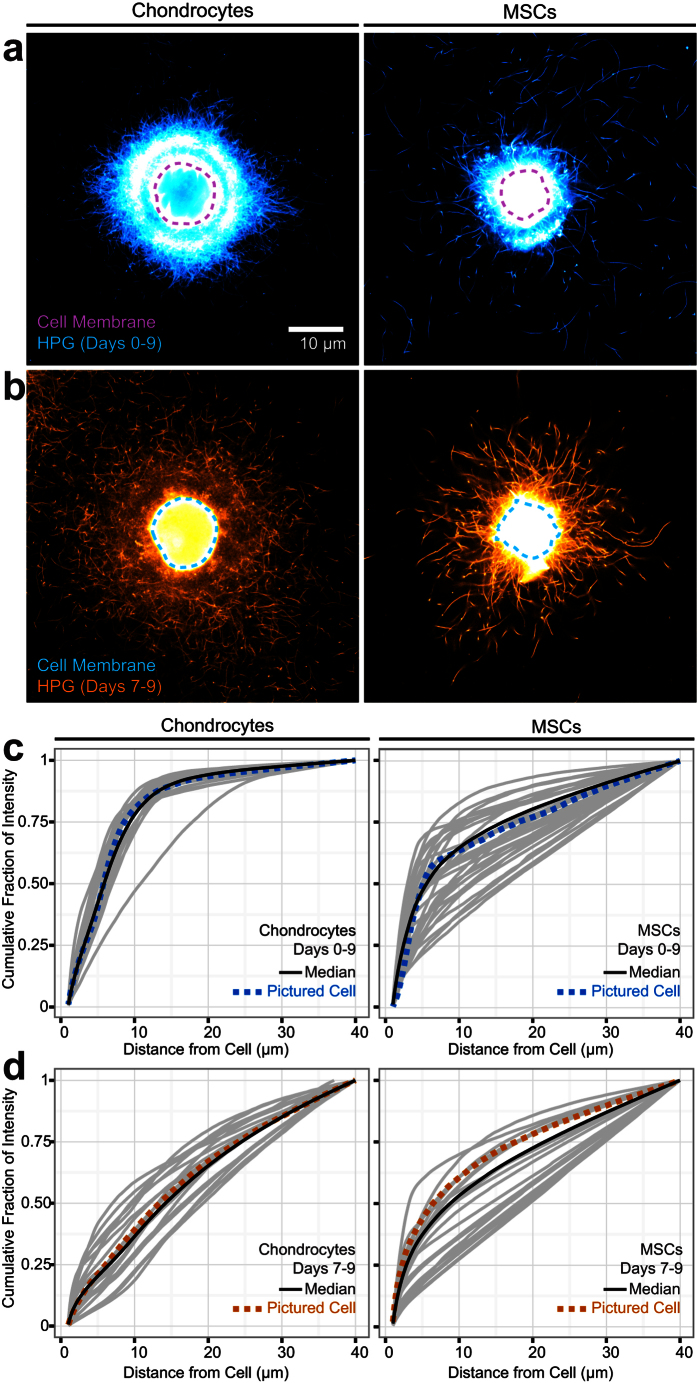
Apparent heterogeneity in matrix accumulation is cell type and timescale dependent. (**a,b)** Representative chondrocytes and MSCs labeled with HPG continuously (a; blue) or between days 7–9 (b; orange). Pulse labeled samples were imaged with higher gain settings than continuously labelled samples, given that the shorter labeling period results in much lower overall intensity (see Fig. S7 for direct comparison). **(c,d)** Quantified matrix distribution of individual cells, represented as the cumulative fraction of total intensity versus distance from the cell. Median is shown in black; representative cells from (**a**) and (**b**) are shown in dashed blue and orange lines. Other individual cells are represented in grey (n = 20 cells/group).

Having shown that chemical manipulation of matrix crosslinking fundamentally altered the distribution of extracellular matrix proteins, we next wondered if FUNCAT labeling could also detect phenotypic differences between cell types that would be reflected in their nascent matrix production and assembly. For example, using standard histological techniques and biochemical assays, we have previously shown that chondrocytes and mesenchymal stem cells (MSCs) differentially produce and organize their ECM^21^. To examine this phenomenon with higher fidelity and with single cell resolution, we next cultured MSCs and chondrocytes in the presence of HPG. Continuous labeling between days 0–9 showed that matrix protein accumulation by MSCs lagged behind that of chondrocytes, and differed in its spatial organization. Notably, there was high MSC-to-MSC variation in the labeled extracellular protein present (Figs 6a,c and S8a,b), consistent with the heterogeneous nature of this cell population^22^,^23^,^24^. This heterogeneity manifested in two ways: in the intensity of the labeled matrix proteins and in the pattern of matrix protein distribution. Select MSCs produced labeled fibrous proteins by day 9, but these molecules were often found far away from the cell, suggesting that MSCs may be less able than chondrocytes to sequester ECM within the pericellular space, or may have a higher level of matrix degradation and turnover. MSCs also sometimes distributed extracellular proteins asymmetrically around the cell body (45% symmetric, 42% asymmetric, 13% without matrix at Day 9; Fig. S8a). In contrast, the organization of continuously labeled matrix protein was very similar between chondrocytes, and was symmetric around individual cells (Fig. S6a). Consistent with continuous labeling, MSCs that were pulse labeled between days 7–9 showed high cell-to-cell variability in matrix accumulation patterns (Figs 6b,d and S8b). Intriguingly, nascent matrix proteins in pulse labeled chondrocytes were markedly heterogeneous (Figs 6b,d and S6b) during this time point. These data indicate that, as the timescale considered shortens, apparent heterogeneity in matrix protein production and organization increases. Taken together, these observations suggest that continuous and pulsed HPG labeling can capture metabolic and phenotypic differences that may be of biological significance in the assembly of the cellular microenvironment at the single cell level.

## Discussion

In this work, noncanonical amino acid tagging revealed cell-, time- and microenvironment-dependent patterns in extracellular matrix assembly and organization. Specifically, we considered the role of these factors in a chondrogenic context, with a focus on how native chondrocytes and differentiating MSCs establish and respond to the cellular niche. Visualization of detailed matrix structures highlighted the differing nature of the proteinaceous ECM produced by these two cell types. Chondrocytes produced well-organized, mechanically-robust matrix proteins that formed a physical barrier and ultimately restricted molecular mobility within the extracellular space. Interestingly, pulse labeling revealed persistent heterogeneity in the active remodeling of this network, which summated to a consistent profile of matrix protein distribution. In contrast, MSCs produced matrix proteins that were loosely organized, poorly retained and often asymmetrically distributed around individual cells. Similarly, on an individual cell basis, nascent matrix proteins produced by MSCs remained heterogeneous in both amount and distribution. Such differences in matrix protein organization likely impact not only the bulk mechanics that develop over time within the construct, but also, and perhaps more importantly, the biochemical and biophysical environment perceived by individual cells at a given time point. ECM organization modulates microscale transport properties, including the mobility and availability of soluble molecules such as growth factors^25^. Furthermore, matrix organization and connectivity influence how forces are transduced to the cell^26^. Thus, the collective organization of matrix proteins is both a key product and determinant of cell phenotype, and is uniquely visualized by FUNCAT labeling.

Both construct maturity and biomaterial density regulated the distribution of nascent matrix proteins in the extracellular space. As the permissivity of the microenvironment decreased, nascent proteins were retained more closely to the cell. That is, the more permissive the microenvironment, the more readily newly-formed matrix could disperse throughout the construct. This finding may explain, in part, why the intentional removal of proteoglycans from developing cartilage constructs hastens subsequent construct growth^27^. Perhaps by decreasing the density of the pericellular microenvironment, mild digestion creates space for increased collagen deposition. Similarly, microenvironmental permissivity may play a role in matrix biosynthesis following injury and during degeneration. In the degenerative condition of osteoarthritis, the density of the extracellular matrix decreases, weakening its mechanical integrity, particularly in the pericellular space^28^. At the same time, osteoarthritic cartilage has higher matrix synthesis activity than healthy tissue^29^. Thus, not only will osteoarthritic cells produce more nascent matrix, but this matrix will have greater mobility in less dense, degenerating tissue (consistent with observations of expanded pericellular matrix in degenerative cartilage^28^).

The utility of FUNCAT labeling also extends to the examination of how chemical and biological perturbations influence matrix assembly and organization. Previously, BAPN treatment of cartilage explants and alginate hydrogels indicated a reduction in the stable incorporation of nascent collagen into the tissue ECM or gel microenvironment^30^,^31^. Here, our results build on these existing bulk analyses and show that this defect in crosslinking manifests organizationally, with a buildup of matrix proteins in the immediate pericellular space, and a decrease in protein content further removed from the cell. It is possible that, upon BAPN washout, this pool of unincorporated collagen becomes available for rapid cross-linking and network assembly, potentially explaining why BAPN pre-treatment can improve integration strength between two pieces of cartilage^31^. Strikingly, fibrous matrix proteins organized perpendicularly to the cell membrane in both control and treated conditions. In tendon cells, the secretion of aligned matrix is a force-dependent process, requiring cellular contractility and a competent cytoskeleton^32^. Our finding that BAPN localized matrix proteins, but inhibited their wider distribution, may suggest that collagen crosslinking is required to establish a functional framework against which newly formed matrix molecules are ‘pushed’ progressively further from the cell.

Modulation of cartilage cross-linking is only one of many possible matrix assembly perturbations. A similar labeling strategy could be used to finely assess the role of specific matrix components; for example, how does knockdown or knockout of individual proteoglycans or structural matrix proteins influence the organization and timing of matrix assembly? Notably, a FUNCAT-based approach would also be able to identify contexts in which turnover is altered, but where total matrix protein content remains unchanged – a situation that traditional staining procedures would be unable to detect. This investigation of the biosynthetic response to injury, disease, or altered genetic program could be performed either *in vitro* (as we assessed tissue engineered construct formation here) or *in vivo*. Previous studies have examined protein synthesis in drosophila and zebrafish systems and mice^14^,^15^,^33^; scaling FUNCAT to mammalian systems with *in vivo* models of cartilage degeneration (e.g. meniscus destabilization) or joint development could lend insight into how matrix proteins form and reorganize during these processes. Recent studies indicate that HPG and AHA can incorporate into developing murine embryos^33^, supporting the potential scalability of FUNCAT analysis to such model systems.

An important limitation of the FUNCAT procedure is that labeling is restricted to methionine-containing proteins. The vast majority of ECM proteins contain methionine: across a set of 361 standard ECM proteins, 85% contain >1% methionine, and 30% contain >2% methionine (Fig. 1a and Table S1). Comparatively, the proteins most commonly found in the cartilage-like matrix (i.e. collagen II and aggrecan) are methionine-poor. Given that we were able to visualize, with high fidelity, ECM predominantly composed of these two molecules, FUNCAT should also be amenable to the examination of the ECM in other tissues, including those rich in fibronectin (1.1% Met), laminins (0.9–2.4% Met), and other collagens (0.6–3.1% Met). Indeed, proteomic analysis has indicated successful *in vivo* incorporation of AHA and HPG across tissues including heart, lung, brain, muscle, and kidney^33^. A notable exception is the protein elastin, which contains only a single methionine residue; such low methionine content suggests that AHA- or HPG-based FUNCAT would be unable to fully detail matrix protein dynamics in elastin-rich tissues (e.g. arteries)^34^. Similarly, FUNCAT is also unable to directly capture the dynamics of non-proteinaceous ECM components, including glycosaminoglycans. Such components may be amenable to assessment through similar metabolic labeling strategies that incorporate azide-modified sugars to monitor nascent glycan synthesis^35^,^36^,^37^,^38^.

A second limitation of the FUNCAT procedure described herein is its reliance on copper to catalyze the azide-alkyne reaction. Copper-catalyzed reactions are well-suited to fixed samples, such as those examined here, but have more limited utility in live-cell imaging applications, where metal toxicity impairs cell viability^39^. A cell-compatible alternative, copper-free click chemistry, relies on strain-promoted cycloaddition between a biomolecule containing azide (e.g. AHA) and cyclooctynes conjugated to fluorophores or affinity tags. Direct comparisons of copper- and copper-free click chemistries in proteomic applications suggest that the copper-free chemistry has decreased sensitivity and increased non-specific labeling in comparison to copper-catalyzed reactions^40^,^41^. Alternatively, the modification of copper-catalyzed click reactions to include ligand acceleration^40^,^42^ or copper chelation^43^ has recently improved cytocompatability to levels comparable with the copper-free chemistry, albeit with modified or additional reagents. Thus, the choice of specific cycloaddition chemistry will require one to establish an application-specific balance between desired biocompatibility, sensitivity, and reaction complexity.

In conclusion, our findings highlight that the spatial organization of extracellular proteins is a sensitive readout modulated by diverse stimuli. While our work focuses on chondrogenesis, the metabolic labeling approach used here offers the ability to query extracellular matrix formation by different cell types, in many different contexts, including tissue degeneration, wound healing, fibrosis, and other pathologic changes in tissue phenotype. Most importantly, given the fidelity and localization of this labeling, these alterations in normal tissue homeostasis can be examined at the single cell level, where such global changes in tissue structure and function first originate. Future applications of this technique will elucidate the spatiotemporal aspects of proteinaceous matrix assembly and remodeling, and contribute to our understanding of how dynamic cell-matrix interactions regulate tissue formation, degeneration, and repair.

## Methods

### Quantifying protein methionine content

Using the UniProtKB/Swiss-Prot database^44^, human proteins of interest were identified using the gene ontology terms ‘extracellular matrix structural constituents’ (GO:0005201) and ‘proteinaceous extracellular matrix’ (GO:0005578). Methionine content was calculated by counting the number of methionine residues relative to the number of total amino acids in each protein sequence.

### Construct fabrication

MSCs and chondrocytes were isolated from juvenile bovine knees (Research 87, Boylston MA) and passaged once before encapsulation in 3D gels. For all conditions, cells were encapsulated at a density of 1.5–2 × 10^6^ cells/mL. To obtain 1%, 2% and 3% (w/v) agarose gels, molten 4% agarose was combined with a warm cell suspension and solidified into thin sheets (~400 μm thick) underneath coverslips^22^. 4 mm × 5 mm μ-gels were cut from the sheets and cultured individually in 48 well plates.

### Functional, noncanonical amino acid labeling

Samples were cultured in a chemically defined media: high glucose DMEM without glutamine, methionine, or cystine (Life Technologies 21013024) supplemented with either 0.1 mM L-methionine (Sigma M5308; control media) or 0.1 mM L-homopropargylglycine (Molecular Probes C10186; labeling media), 10 ng/ml TGFβ-3, 0.1 μM dexamethasone, 4 mM L-glutamine, 0.201 mM cystine (Sigma C7602), 100 μg/mL sodium pyruvate, 1.25 mg/mL bovine serum albumin, 0.1% ITS premix, 50 μg/mL ascorbate 2-phosphate, 40 μg/mL proline, and 1% penicillin-streptomycin-amphotericin. Medium was replenished every 2–3 days, and samples were finally fixed in 4% phosphate-buffered paraformaldehyde for 15 minutes before storage in PBS at 4 °C. Cartilage plugs (4 mm diameter) were cultured in labeling media for 3 days, and fixed in 4% PFA overnight.

HPG incorporated into fixed samples was covalently tagged with Alexa Fluor 488. Samples collected up to day 9 were stained and imaged as intact gels. To improve imaging, cartilage and gels collected at 21 and 42 days were cryosectioned to yield 40 μm thick cross-sections. Both intact gels and cryosections were first stained with a 1:1000 dilution of a plasma membrane stain (Molecular Probes C10046) in PBS for 30 minutes at room temperature. Next, samples were rinsed twice with PBS, and incubated in a click reaction labeling solution (prepared from Molecular Probes C10428 according to product instructions, and including Alexa Fluor 488 azide) for 40 minutes at room temperature. Samples were washed in reaction rinse buffer (Molecular Probes C10428, 5 minutes at room temperature), and then once with PBS. Nuclei were labeled with Hoechst in PBS for 15 minutes at room temperature, and samples were washed twice with PBS before imaging. The same labeling procedure was followed for AHA samples, with Alexa Fluor 594 alkyne (Molecular Probes C10102 and A10275).

### FUNCAT Imaging and Image Quantification

Samples were mounted in PBS and imaged using a Nikon A1 confocal microscope. To acquire large fields of view (Fig. S1), confocal slices were captured at 40X. To visualize staining near individual cells, cells within 100 μm of the gel surface were identified via nuclear staining, and 100X confocal sections were taken through the cell midplane. To quantify the staining associated with each cell, 20 radial intensity profiles emanating from the cell center were mapped, truncated to include only the extracellular domain (demarcated by membrane staining), and averaged over each cell. Any encroaching matrix from nearby cells was manually excluded from this quantification. For easier visualization, post-processing was used to enhance the contrast; all images in a subfigure were imaged and post-processed identically. Quantified HPG intensity values shown in graphs were not transformed and are comparable between Figs 3 and 5A. Sample size was selected based on a bootstrap resampling of a dataset of n = 40 cells; samples sizes of n = 5, 10, 20, 30 and 40 cells were simulated over 100 bootstrap replicates. Aggregate metrics were near-identical between n = 20, 30, and 40 cells, suggesting that a sample size of n = 20 cells per condition balanced sufficient statistical coverage with experimental efficiency.

To determine matrix radius, background signal levels were determined for each profile. Radius was considered as the first distance where intensity dipped below 110% of the background signal level. Profiles were averaged to determine the matrix radius of each cell; values reported are the mean ± standard error of 20 cells.

### Alcian Blue Staining and Immunofluorescence

Histological sections or intact μ-gels were stained with Alcian blue pH 1.0 (Rowley Biochemical) to identify sulfated proteoglycans^22^. Immunofluorescence staining was performed following the cell membrane, HPG and nuclear staining described above. μ-gels were stained for aggrecan (1°: Abcam ab3778, 1:50 in PBS overnight at 4 °C; 2°: ThermoFisher AlexaFluor 546 goat anti-mouse, 1:200 in 5% BSA for 1 hr at room temperature). Additional μ-gels were stained for collagen II (1°: DSHB ii-ii6b3, 10 μg/mL in PBS overnight at 4 °C; 2°: ThermoFisher AlexaFluor 546 goat anti-mouse, 1:200 in 5% BSA for 1 hr at room temperature). Collagen II staining was performed with and without hyaluronidase digestion (300 μg/ml, 2.5 hours at room temperature).

### Matrix Digestion and Perturbation

Following 9 days of continuous culture in labeling media, agarose μ-gels were digested with a panel of enzymes. Highly purified collagenase (Worthington CLSPA) was suspended at 300 U/mL in PBS. Chondrotinase ABC (Sigma C2905) was suspended at 0.4 U/mL in 50 mM Tris, 60 mM sodium acetate, 0.02% BSA, pH 8.0. Hyaluronidase (Type IV-S from bovine testes, Sigma H3884) was suspended at 300 μg/mL in PBS. Digestion was performed at 37 °C for either 1 or 18 hours. To assess the impact of a decrease in collagen crosslinking, β-aminopropionitrile (BAPN, Sigma A3134) was added to labeling media at 300 μM^45^. BAPN was applied for varying time periods as indicated in the results and figure legends.

### Microcompression

Agarose gels seeded with 2 × 10^6^ chondrocytes/mL were cast between two glass plates and cut to yield cylindrical samples measuring 4 mm in diameter and 2 mm thick. Constructs were cultured in either control media (native methionine) or labeling media (HPG) for up to 9 days. Unfixed samples were halved through the mid-sagittal plane and stained with calcein AM and Hoechst to mark cell bodies and nuclei. Using a confocal-mounted device^46^, samples were compressed to 30% strain in increments of 5%. Before each compressive step, 3D stacks depicting a region of interest were imaged. Following each compressive step, the sample was allowed to equilibrate for 5 minutes. Individual cells depicted in stack maximum Z projections were computationally segmented and manually tracked through multiple strain levels. For identified cells, the cell aspect ratio was calculated as the ratio of the long axis to the short axis. Data are presented as the ratio of strained to unstrained cell aspect ratio, calculated on a single cell basis.

## Additional Information

**How to cite this article**: McLeod, C. M. and Mauck, R. L. High fidelity visualization of cell-to-cell variation and temporal dynamics in nascent extracellular matrix formation. *Sci. Rep.* **6**, 38852; doi: 10.1038/srep38852 (2016).

**Publisher's note:** Springer Nature remains neutral with regard to jurisdictional claims in published maps and institutional affiliations.

## Supplementary Material

Supplementary Information

Supplementary Table S1

Supplementary Video S1

Supplementary Video S2
