# Supplementary material for: High fidelity visualization of cell-to-cell variation and temporal dynamics in nascent extracellular matrix formation
Source: Sci Rep. 2016 Dec 12;6:38852. doi: 10.1038/srep38852 (PMC5150986; doi:10.1038/srep38852)
Supplement: Supplementary Information [file srep38852-s1.pdf]

Supplementary Info:

High fidelity visualization of cell-to-cell variation and temporal dynamics in nascent extracellular matrix formation

Claire M McLeod[1,2,3] & Robert L Mauck[1,2,3*]

[1] Department of Bioengineering, University of Pennsylvania, Philadelphia, PA 19104, USA
[2] McKay Orthopaedic Research Laboratory, Department of Orthopaedic Surgery, Perelman School of Medicine, University of Pennsylvania, Philadelphia, PA 19104, USA
[3] Translational Musculoskeletal Research Center, Philadelphia VA Medical Center, Philadelphia, PA 19104, USA.

***Contact information:**
Robert L. Mauck, Ph.D.
McKay Orthopaedic Research Laboratory
Department of Orthopaedic Surgery
University of Pennsylvania
Philadelphia PA 19104
T: 215-898-3294; F: 215-573-2133
lemauck@mail.med.upenn.edu

Movie S1: 3D reconstruction of the nascent matrix formed by a chondrocyte continuously labeled with HPG for 9 days. Reconstruction depicts a 5 μm thick confocal stack, colored by depth, taken near the cell midplane. Fibrous structures project outwards from the cell and extend through multiple z-planes.

Movie S2: 3D reconstruction of the nascent matrix formed by a chondrocyte continuously labeled with HPG for 9 days. Reconstruction depicts a 5 μm thick confocal stack, colored by depth, taken below the cell body. Fibrous structures form a mesh network, and many are vertically oriented (emanating from the cell body).

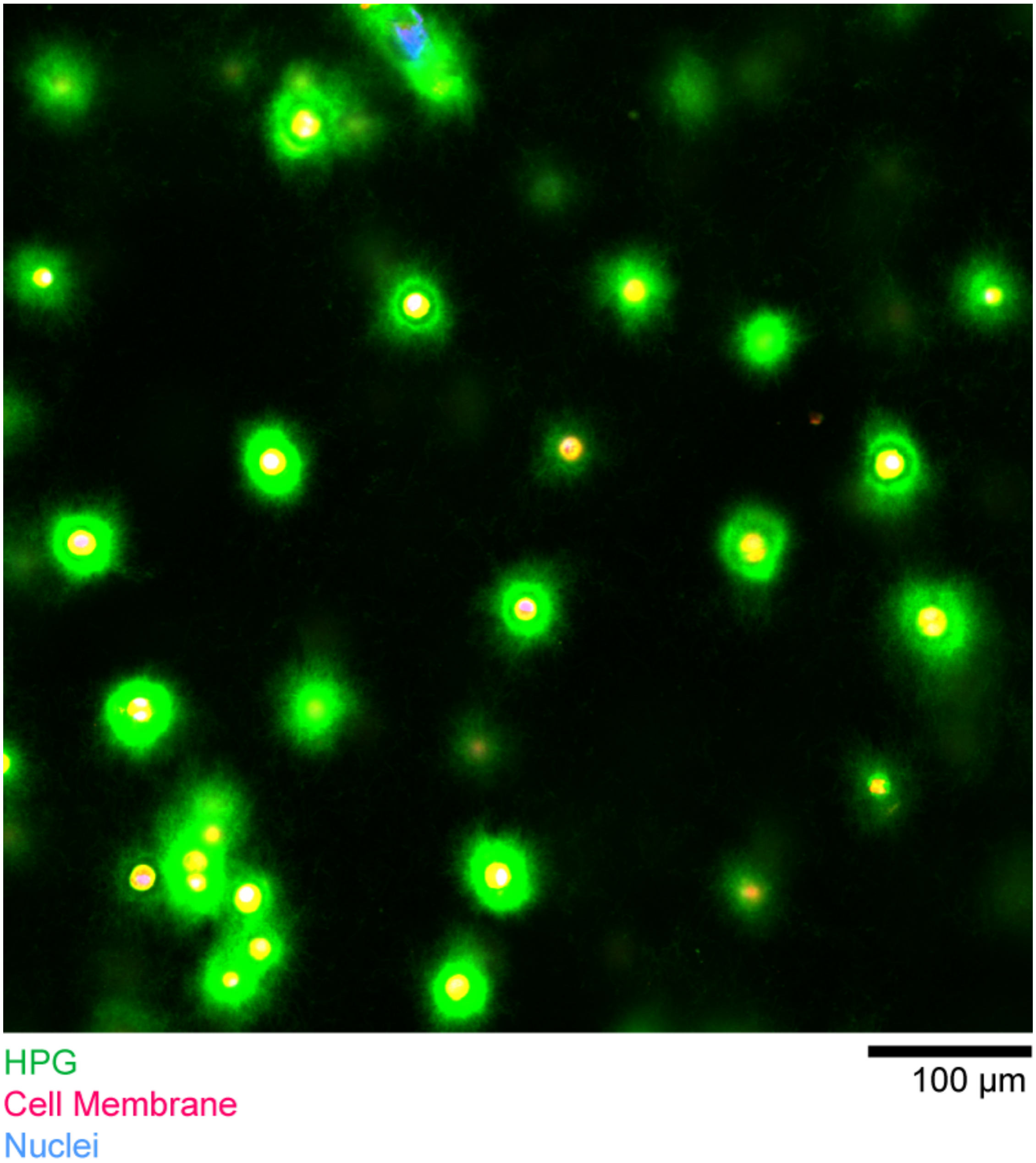

Figure S1: Large area scans confirm uniform labeling across gel. Chondrocytes in agarose, cultured in HPG labeling media for 9 days. Scan taken at 40X, ~ 50 μm into the gel.

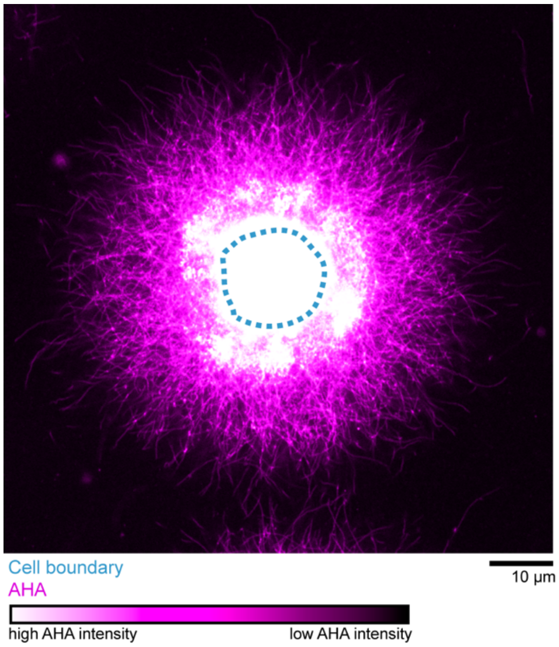

Figure S2: Matrix labeling with azidohomoalanine (AHA). Mid-plane confocal cross-section of a chondrocyte cultured in AHA labeling media for 7 days and tagged with Alexa Fluor 594-alkyne.

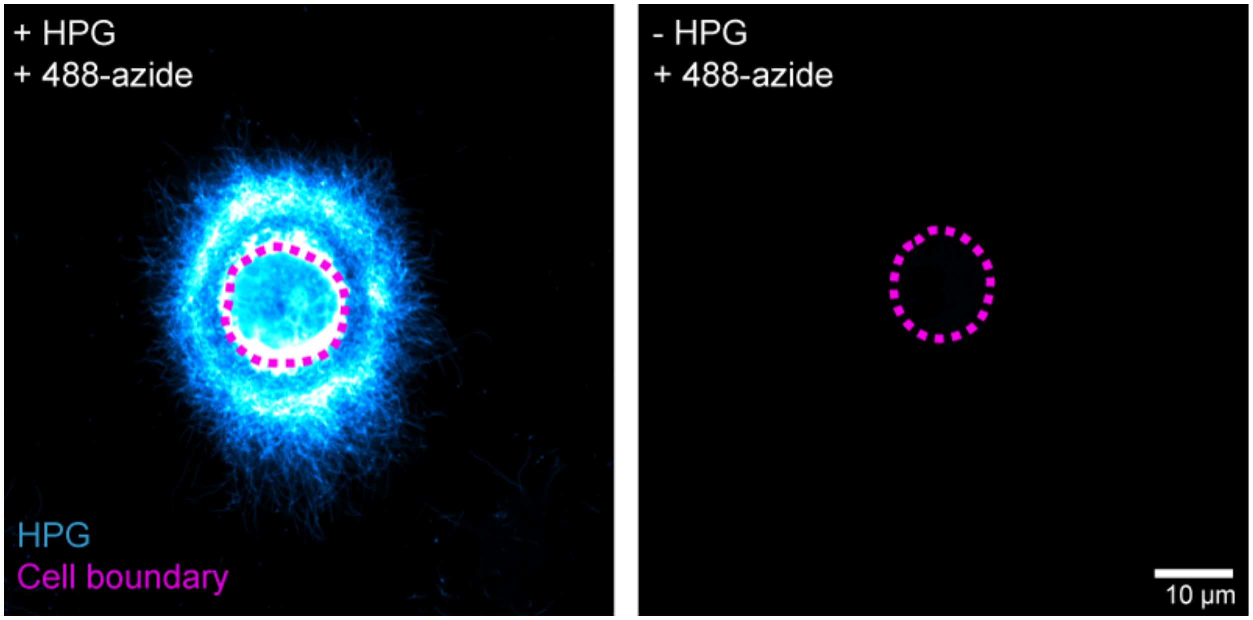

Figure S3: Fluorescent tagging of HPG is highly specific. Mid-plane confocal cross-sections of two chondrocytes, cultured in either labeling media (with HPG, left) or control methionine media (no HPG, right) for 9 days, and fluorescently tagged with 488-azide.

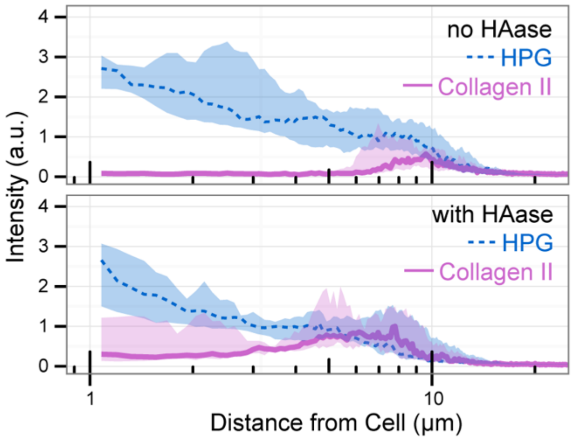

Figure S4: Hyaluronidase digestion of fixed samples increases collagen II detection by immunofluorescence, but does not alter HPG labeling. Radial intensity profiles for chondrocytes cultured in agarose and labeled with HPG for 9 days. Following fixation, samples were incubated in either PBS (control) or hyaluronidase before immunostaining and HPG tagging. Lines represent median intensity profile, shaded areas represent 25th to 75th percentiles (n = 9-11 cells/group).

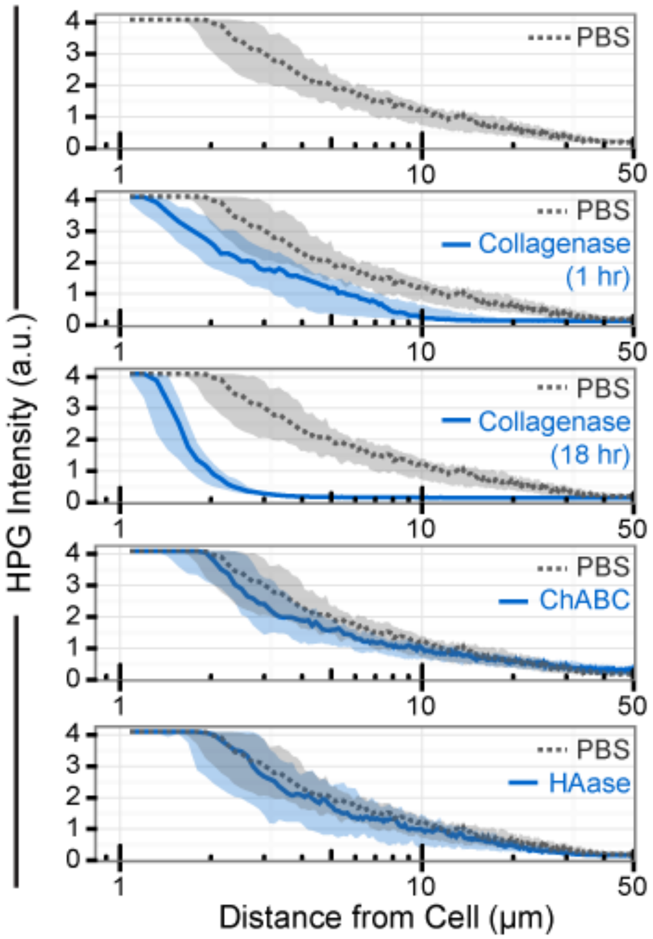

Figure S5: Additional plots quantifying enzymatic of HPG-labeled matrix. Data from Fig 2c, re-visualized with log(distance) on the x-axis. Radial profile quantification of HPG intensity following digestion, compared to constructs incubated in PBS. Lines represent median intensity profile, shaded areas represent 25th to 75th percentiles (n = 20 cells/group).

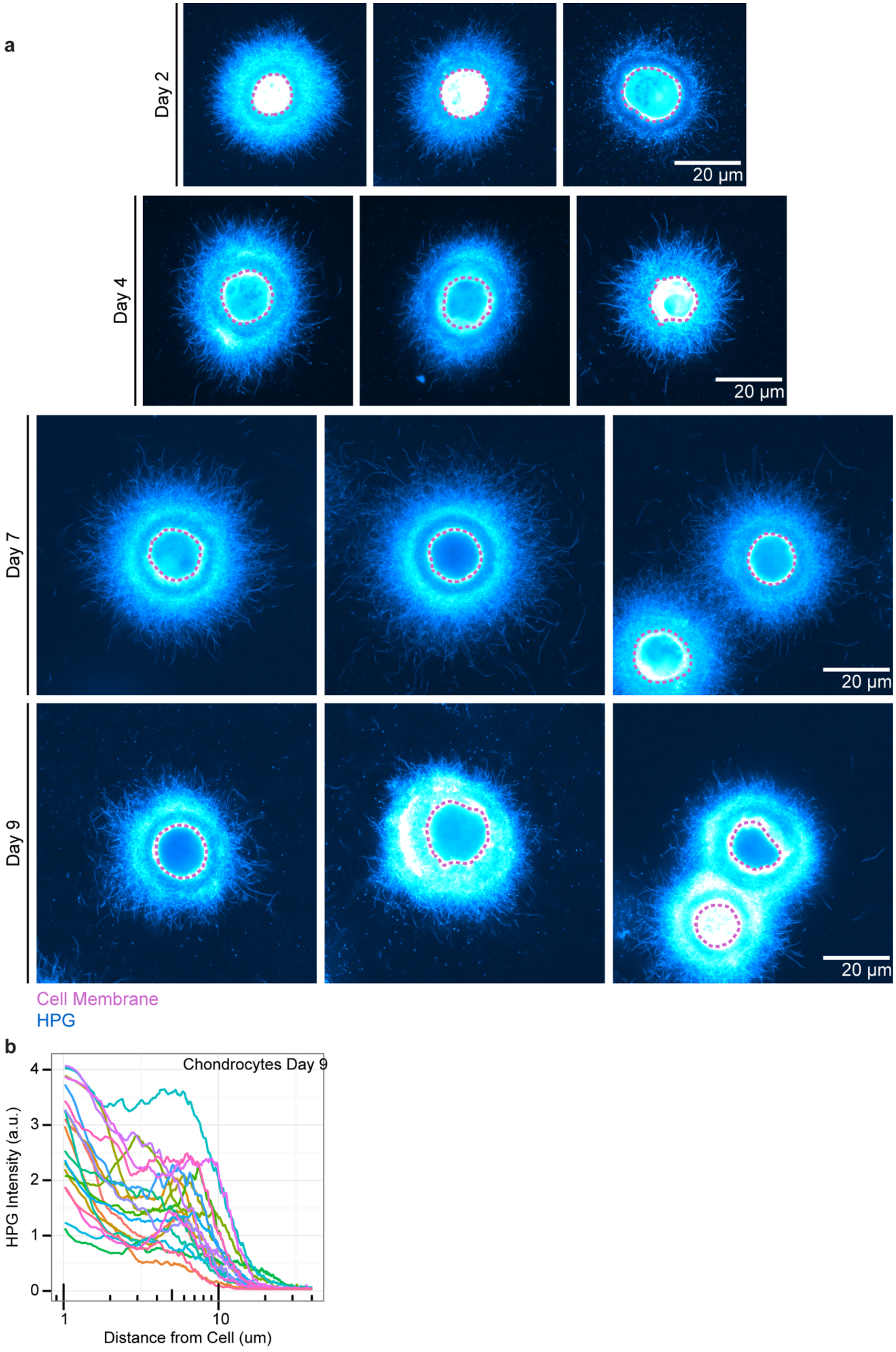
a
Day 2
20 µm
Day 4
20 µm
Day 7
20 µm
Day 9
20 µm
Cell Membrane
HPG
b
Chondrocytes Day 9
HPG Intensity (a.u.)
0
1
2
3
4
1
10
Distance from Cell (um)

Figure S6: Cell-to-cell heterogeneity in HPG-labeled nascent matrix formed by chondrocytes. (a) A panel of representative chondrocytes cultured in agarose and labeled with HPG for up to 9 days. Colormap and scale bar are consistent across all images. **(b)** Radial intensity profiles for 20 individual chondrocytes cultured and labeled for 9 days.

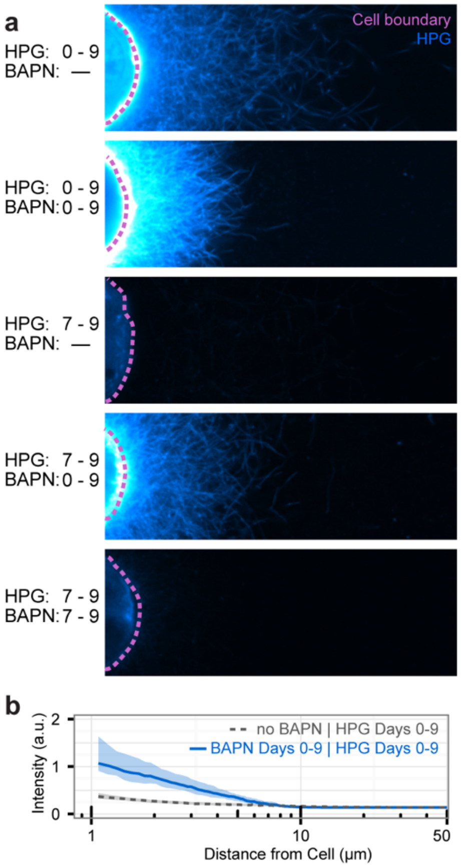

Figure S7: Exposure to BAPN increases nascent matrix synthesis. (a) Continuous and pulse labeling (final 2 days) of chondrocytes cultured in the presence and absence of BAPN, a collagen cross-linking enzyme inhibitor. Pulse labeled cells are the same cells shown in Figure 5, visualized here using the same imaging settings as the continuously labeled groups. **(b)** Radial intensity profiles of chondrocytes continuously labeled with HPG for 9 days in the presence and absence of BAPN. Lines represent median intensity profile, shaded areas represent 25th to 75th percentiles (n = 20 cells/group).

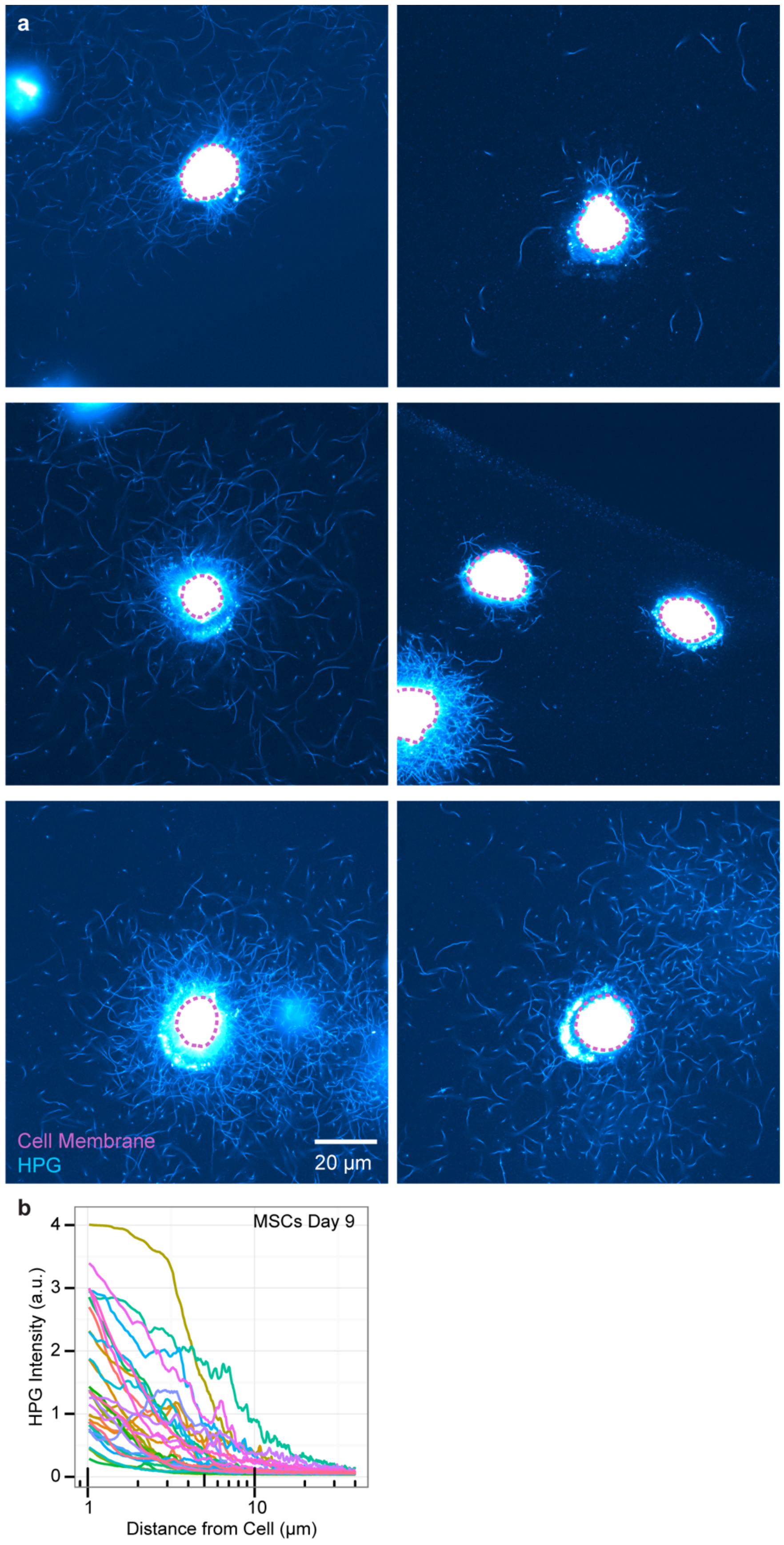
a
Cell Membrane
HPG
20 µm
b
MSCs Day 9
HPG Intensity (a.u.)
Distance from Cell (µm)
4
3
2
1
0
1
10

Figure S8: Cell-to-cell heterogeneity in HPG-labeled nascent matrix formed by mesenchymal stem cells (MSCs). (a) A panel of representative MSCs cultured in agarose and labeled with HPG continuously for 9 days. **(b)** Radial intensity profiles for 20 individual MSCs cultured and labeled for 9 days.
